# Editorial: Neuroendocrine signalling pathways along the microbiota-gut-brain axis in functional gut disorders

**DOI:** 10.3389/fendo.2022.996382

**Published:** 2022-09-21

**Authors:** Maria Cecilia Giron, Andreina Baj, Cristina Giaroni

**Affiliations:** ^1^ Department of Pharmaceutical and Pharmacological Sciences, University of Padova, Pharmacology Building, Padova, Italy; ^2^ Department of Medicine and Surgery, University of Insubria, Varese, Italy

**Keywords:** stress, visceral pain, functional gastrointestinal disorders, microbiota-gut-brain axis, irritable bowel syndrome (IBS)

Irritable bowel syndrome (IBS) is the most common among functional gastrointestinal disorders (FGIDs) characterized by non-structural symptoms that destabilize the patient’s quality of life with increasing incidence in industrialized countries. Although the exact etiopathogenesis remains unknown, FGDIs develop as a consequence of miscellaneous factors, such as genetic, environmental, immune, inflammatory, neurological and psychological triggers, in addition to visceral hypersensitivity and postinfectious events. The interplay between an altered gut physiology and psychological factors is suggested to depend upon the existence of bidirectional communication pathways where the gut and the brain influence each other along the so-called “gut-brain axis”. This communication axis involves the gut microbiota, which plays a pivotal role in maintaining local, systemic and gut-brain homeostasis, by secreting several neuroactive molecules, hormones and metabolites. From the seminal discovery, about 30 years ago, of noradrenaline and adrenaline as neuroendocrine molecules along the microbiota-gut-brain axis, it is now ascertained that several other neuroactive molecules are produced by microbes inhabiting the human gut. Tryptophan-derived metabolites, such as serotonin (5-hydroxytryptamine (5-HT)), kynurenine and indoles, neurotransmitters, such as glutamate, γ-aminobutyric acid (GABA) and acetylcholine, neuroactive short- and medium-chain fatty acids, secondary bile acids, branch-chain aminoacids are among the most relevant molecules produced by saprophytic bacteria (1). The present Research Topic highlights the more recent advances on the involvement of neuroendocrine signalling pathways along the microbiota-gut-brain axis in the pathogenesis of FGIDs.

In this context, the review of Layunta et al. defines 5-HT, a fundamental neurotransmitter/neuromodulator involved in the control of both gut physiology and central nervous system responses, as a key molecule contributing to microbiota-gut-brain axis dysfunction related to FGIDs. The involvement of serotoninergic pathways in IBS pathogenesis is well ascertained and clinical studies suggest that the modulation of both serotonin reuptake transporter (SERT) activity and 5-HT metabolism represent potential therapeutic approaches. More importantly the efficacy of treatments aiming at regulating the serotoninergic system is improved by the modulation of the microbiota. In this latter context gut microbes by controlling the metabolism of tryptophan, the precursor of 5-HT, may influence the gut and brain levels of this neuroamine (2). Furthermore, pattern recognition receptors, recognizing key microbial sensors involved in the innate immune response, such as Toll-like receptors (TLRs) and Nucleotide oligomerization domain (NOD)-like receptors (NLRs), have a critical role in altering serotoninergic tone and vice versa (3). Indeed, changes in their expression may represent a predictive marker for the development of IBS as well as brain disorders. The possibility to influence the homeostasis of neuroactive molecules such as 5-HT and GABA by means of microbiota-based treatment to attenuate gut and central nervous system symptoms related to IBS, has been extensively described in the review by Chen et al., which underlines the potential of dopamine and histamine as additional amine neurotransmitters that have received limited attention until now (4). However, manipulation of the indigenous microbiota has been shown to be an effective approach primarily on animal-based investigations since clinical evidence is still quite poor with variable results on disease outcomes mainly due to patient heterogeneity (5). Neuroendocrine signals involving hormones, such as sex steroids, along the microbiota-gut brain axis may also have a pathogenetic role in IBS and contribute to the sexual dimorphism, which represents a main feature of IBS, with females more prone to develop the disorder with respect to males. This topic is developed in the review by So and Savidge, which emphasizes the contribution of both sex steroids and gut microbiota to the development of visceral, motor and immune responses in IBS. The established relationship between sex steroids and enteric bacteria leads to the hypothesis that gut microbes may influence sex hormone effects. From a mechanistic viewpoint, which, however, still needs to be fully proven, the interplay between sex steroids and gut microbiota in IBS may involve activation of epigenetic changes on neuronal and immune pathways. Indeed, a clear-cut correlation between sex hormones, their interaction with the gut microbiota and IBS development still needs to be confirmed by more consistent preclinical and clinical data based on stratified trial design by sex, age and more standardized treatments.

A dysfunctional gut-brain axis in FGIDs may involve local paracrine and neurocrine factors such as bile acids, as detailed in the review article by Dhonnabháín et al. Besides being fundamental for digestion and lipid absorption, bile acids may influence the composition of bacterial communities by exerting a potent anti-microbial activity. Furthermore, several bile salt hydrolase-containing bacteria favor the transformation of primary bile acids into secondary bile acids, enabling further microbially mediated conversion to produce a myriad of secondary bile acids, shaping the bacterial profiles within colonic microbiome. By binding to bile acid receptors (e.g., FXR, TGR5), expressed on 5-HT-secreting enterochromaffin cells, GLP-1-secreting L-cells, immune cells and neural cells, bile acids exert a modulatory role on intrinsic and central neuronal pathways controlling sensory and motor gut functions. Preclinical and clinical studies suggest a role of bile acids in the pathogenesis of IBS. The striking relationship between prokinetic actions of bile acids on colonic function and the occurrence of diarrhea-associated IBS symptoms supports interventions aiming at modifying luminal exposure to bile acids as therapeutic approaches for FGIDs treatment.

Overall, the correlation between gut dysbiosis, dysmotility and neurologic/psychological symptoms related to FGIDs has risen the interest in the supplementation of a biotic-based therapy by means of prebiotics, probiotics, fermented foods or synbiotic and faecal microbiota transplantation, to restore intestinal homeostasis. However, as critically discussed in the review of Reid et al. the efficacy of these approaches is still under debate, and the impact of specific strains and related metabolites in the gut and in extraintestinal tissues, such as the brain, has not yet been fully clarified. Since novel biotic-based strategy are emerging, the application of diverse genetic, microbiological and molecular imaging techniques in more rigorous preclinical studies will highly contribute to identify valuable target strains together with the effective activity of their metabolites to influence the enteric, peripheral and central nervous system function, before entering more expensive and large clinical studies.

Despite these evident limitations, articles of this Research Topic converge on the importance of recognizing those saprophytic bacterial strains which have more profound effects on the host than others, although it is now clear cut that there won’t be any magic-bullet strains that work for every patient. Currently, a major challenge going forward will be to decipher the neuroendocrine signaling pathways along the microbiota-gut-brain axis for finding subtle and safe strategies to target both microbiota dysbiosis, metabolic changes and neuroimmune dysfunctions to treat FDGIs and prevent their relapses.

## Author contributions

AB and MG contributed to the design of the work and revised it critically. CG contribute to the design and wrote the manuscript. All Authors provided approval for publication

## Conflict of interest

The authors declare that the research was conducted in the absence of any commercial or financial relationships that could be construed as a potential conflict of interest.

## Publisher’s note

All claims expressed in this article are solely those of the authors and do not necessarily represent those of their affiliated organizations, or those of the publisher, the editors and the reviewers. Any product that may be evaluated in this article, or claim that may be made by its manufacturer, is not guaranteed or endorsed by the publisher.
